# The diversity of STING in regulating immune cell function and its role in liver diseases: from bench to bedside

**DOI:** 10.1186/s13578-026-01622-y

**Published:** 2026-07-10

**Authors:** Jing Lin, Yongle Liu, Yu Chen, Xiaohui Zhang

**Affiliations:** 1 Fourth Department of Liver Disease, Beijing Youan Hospital, Capital Medical University, Beijing, 100069 China; 2 Beijing Key Laboratory of Liver Regeneration and Artificial Liver Transformation Research, Beijing, 100069 China

**Keywords:** cGAS-STING pathway, Liver immunity, Immune cells, Lver diseases

## Abstract

**Background:**

The cyclic guanosine monophosphate-adenylate synthase (cGAS)-stimulator of interferon genes (STING) pathway is a critical innate immune signaling pathway that recognizes and transmits cytoplasmic DNA signals, triggering interferon and inflammatory responses. Immune cells enriched in the liver participate in the development of various liver diseases through the STING pathway; however, the precise regulatory mechanisms of this pathway within the immune cells remain poorly integrated. Elucidating these mechanisms holds promise for developing novel therapeutic strategies to address related clinical challenges.

**Main body:**

This review systematically elucidates the mechanisms by which immune cells from both innate and adaptive immune systems influence liver diseases via the STING pathway, viewed through the lens of immune cell classification. Considering the differential expression of STING across immune cell types and their cross-regulatory interactions, the review categorizes STING’s impact on liver diseases into two patterns: direct regulation by endogenous STING (intracellular STING) and indirect regulation by exogenous STING (STING originating from other cells). The diseases discussed encompass common liver disorders, such as viral hepatitis, metabolic dysfunction-associated steatotic liver disease(MASLD), hepatocellular carcinoma(HCC), and autoimmune hepatitis (AIH),among others.Integrating the latest preclinical research findings, the review thoroughly explores the potential feasibility and research progress of targeting the STING pathway to modulate the progression of liver diseases, including traditional STING agonist/antagonist, and novel approaches such as targeted delivery systems and microbiotherapy in STING drug development, along with their therapeutic potential in liver diseases.

**Conclusions:**

The cGAS-STING pathway serves as a pivotal signaling axis linking innate and adaptive immunity, playing a crucial role in the immune regulation of liver diseases. In-depth investigation of this pathway provides theoretical and translational foundations for elucidating the mechanisms underlying immune-metabolic dysregulation in the liver and developing precision immunotherapy strategies, thereby facilitating its transition from basic research to clinical treatment.

## Molecular structure and functional basis of the cGAS-STING pathway

The cyclic GMP-AMP synthase (cGAS)-stimulator of interferon genes (STING) pathway, discovered at the turn of the century, constitutes an innate immune signaling pathway that plays a pivotal role in host defense and the maintenance of immune homeostasis.The key molecules STING and cGAS within this pathway were successfully identified in 2008 and 2013 respectively. With advancing research, their functions in anti-infective immunity, immune regulation, and regulation of cross-physiological processes have been progressively and systematically elucidated. This provides a core theoretical framework for understanding innate immune signaling mechanisms and the pathogenesis of related diseases.

The molecular structures of cGAS and STING are intricately linked to their functions within the signaling pathway. cGAS, the core cytoplasmic DNA sensor, consists of an N-terminal unstructured region, a central Mab21 catalytic domain, and an extended N-terminal regulatory domain. Under pathological conditions such as viral infections, mitochondrial damage, or genomic instability, dsDNA accumulates abnormally in the cytoplasm [1].Through electrostatic interactions between its N-terminal region and DNA, cGAS captures DNA specifically, releasing its catalytic domain from auto-inhibition and activating its nucleotidyl transferase activity. In this activated state, cGAS catalyzes the production of cyclic GMP-AMP (cGAMP) [2–5].

The downstream protein STING, encoded by the TMEM173 gene, has an N-terminal tetramembrane domain anchored to the endoplasmic reticulum (ER) membrane and a C-terminal cytoplasmic region containing a cyclic dinucleotide- binding domain (CBD) and a carboxyl-terminal tail (CTT). When cGAMP binds to STING’s CBD, it induces STING dimerization and oligomerization, facilitating its translocation from the endoplasmic reticulum to the Golgi apparatus. At the Golgi, the CTT recruits key downstream signaling molecules, such as TANK-binding kinase 1 (TBK1) and the IκB kinase (IKK) complex, activating the interferon regulatory factor 3 (IRF3)-mediated type I interferon (IFN-Ⅰ) pathway and the nuclear factor kappa-B (NF-κB)-mediated pro-inflammatory pathway [6–9].

These two pathways, while relatively independent, also interact through cross-regulatory mechanisms within STING signaling. For example, TBK1 indirectly enhances NF-κB activity via IKKε, and type I interferon(IFN-I) provides feedback enhancement to NF-κB signaling, forming a positive feedback loop that fine-tunes the intensity and timing of the immune response [9, 10]. This precisely regulated mechanism is essential for maintaining immune homeostasis and preventing excessive inflammation. Moreover, STING can recognize not only host-derived cGAMP but also cyclic dinucleotides (CDNs) secreted by various pathogens, such as bacteria, highlighting its broad-spectrum role in cross-species immune defense and its position as a crucial link between innate and adaptive immune responses [7–9]. Overall, the cGAS-STING signaling pathway plays a critical role in both organic self-damage and pathogen infection: it is not only triggered by damaged double-stranded DNA (dsDNA) and cytoplasmic damaged mitochondrial DNA (mtDNA), but is also recognized by bacterial-derived c-di-AMP during infection.

**Fig. 1:**
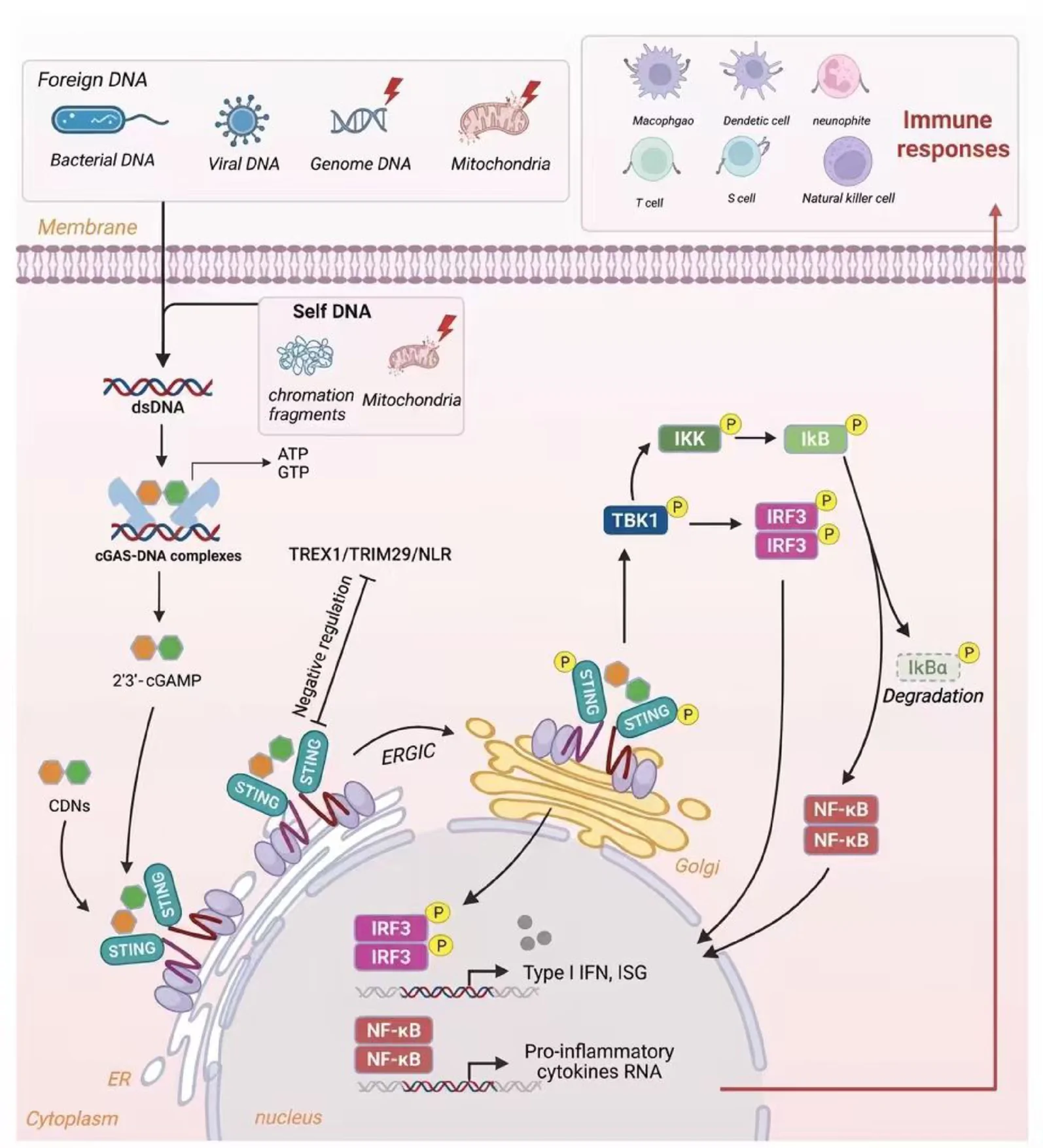
Schematic representat of the cGAS-STING signaling pathway. Cytoplasmic accumulation of double-stranded DNA binds to the N-terminal region of cGAS, activating its nucleoside transferase activity to catalyse the production of the second messenger cGAMP using GTP and ATP as substrates. Upon binding to the CBD domain of STING, cGAMP induces STING dimerization and oligomerization, facilitating its translocation from the endoplasmic reticulum to the Golgi apparatus. This process recruits TBK1 and the IKK complex, activating the IRF3-mediated IFN-I/ISG transcriptional pathway and the NF-κB-mediated pro-inflammatory cytokine expression pathway, respectively. These pathways form positive feedback regulation through TBK1-IKKε interactions and IFN-I feedback, while molecules such as TREX1 negatively regulate pathway activity by degrading substrates and binding key signaling molecules, collectively maintaining immune homeostasis

The body also employs negative regulatory mechanisms, including the three‑prime repair exonuclease 1 (TREX1) that degrades intracellular DNA and components of the ubiquitin-proteasome or lysosomal-autophagic systems that degrade STING, such as TRIM29, RNF5/RNF90, and ATG9a. Additionally, NOD-like receptor (NLR) family proteins can cross-inhibit STING; for instance, NLRC3, NLRX1, and NLRP14 can bind to TBK1 or STING, blocking downstream signaling and exerting negative regulatory effects [11, 12] (Fig. 1). In addition to its role in immune regulation, recent studies have revealed that STING possesses regulatory functions beyond its innate immune-related activities: the pivotal role of STING in the regulation of various forms of cell death, including apoptosis, necroptosis, pyroptosis, ferroptosis, autophagy-dependent cell death (ADCD), and immunogenic cell death (ICD) [13]. These findings highlight the broader significance of STING in cellular physiology beyond its innate immune functions.

## The cGAS-STING pathway and the liver immune system

The liver, as a vital digestive organ in the human body, traditionally fulfills essential physiological functions such as metabolism, nutrient storage, and detoxification [14]. However, recent research has increasingly demonstrated that the liver also acts as a central hub for metabolic and immune regulation [15]. Under physiological homeostasis, the liver’s immune equilibrium primarily relies on the coordinated action of a non-parenchymal cell network. This includes Kupffer cells (KCs), liver sinusoidal endothelial cells (LSECs), dendritic cells (DCs), and natural killer (NK)/natural killer T (NKT) cells. These cells establish a multi-layered immune defense system by recruiting circulating T/B cells, neutrophils, and other cells [16–18]. This highly specialized immune microenvironment confers unique immunoregulatory capabilities to the liver, enabling it to maintain immune tolerance towards harmless portal vein-derived antigens while rapidly initiating immune responses during pathogen invasion or tissue injury. Dysregulation of this hepatic immune control is closely associated with the onset and progression of various liver diseases, including viral hepatitis, metabolic dysfunction-associated steatotic liver disease (MASLD), and hepatocellular carcinoma (HCC) [19–21]. In MASLD, excessive lipid accumulation in hepatocytes induces mitochondrial dysfunction and oxidative stress, leading to hepatocyte injury and death; dying hepatocytes further release damage-associated molecular patterns (DAMPs), such as DNA, which exacerbate inflammation and liver injury.

As a key signaling axis in innate immune responses, the cGAS-STING pathway exhibits distinct cell-type-specific expression patterns within the liver. The majority of immune cells involved in liver immunity, especially resident populations like KCs and NK cell, highly express STING, which plays a critical role in hepatic immune defense and surveillance. This characteristic plays an important role in hepatic immune defense and surveillance [22, 23]. Upon activation of the cGAS-STING pathway in hepatic immune cells, it not only induces moderate autocrine IFN-I expression to enhance immune responses, but also modulates inflammatory responses through intercellular crosstalk affecting other immune cell functions [24], playing a central role in infection defense, tumor immune surveillance, and autoimmune regulation [25, 26]. However, excessive or aberrant activation of this pathway can trigger cytokine storms and tissue damage [27]. This leads to NF-κB-dependent chronic inflammation, fibrosis, and T cell exhaustion, thereby driving disease progression [28].

In sharp contrast, STING is expressed at low levels in hepatocytes [29], which may serve as a self-protective mechanism. As hepatocytes are rich in mitochondria, mitochondrial dysfunction under oxidative stress can lead to mtDNA leakage [30]; low STING expression helps limit such leakage, suppress cGAS-STING-driven inflammation, and sustain cell survival [31]. However, weak STING expression also impairs antiviral immunity. For example, hepatitis B virus evades cGAS-STING surveillance via specific genome packaging, enabling persistent infection [32]. Importantly, hepatocytes are a major source of mtDNA and nuclear DNA. Under pathological stress, damaged or dying hepatocytes release DNA that activates STING in neighboring immune cells [33]. During hepatic ischemia‑reperfusion injury, hepatocyte mtDNA release is significantly increased and potently activates STING signaling in macrophages, triggering caspase‑1/GSDMD‑mediated pyroptosis and liver inflammation [34]. Inhibiting mtDNA release or depleting macrophages effectively attenuates cGAS-STING activation and alleviates hepatic ischemia‑reperfusion injury [33]. The differential STING expression between hepatocytes and intrahepatic immune cells is the core upstream mechanism underlying the contradictory functional outcomes of STING in liver diseases.

This review systematically elucidates the multiple regulatory mechanisms and mediated biological effects of the cGAS-STING pathway across distinct immune cells within the hepatic immune microenvironment. It focuses on both the intrinsic activation (direct regulation) of this pathway and the interactive signaling networks involving multiple immune cells (indirect regulation), alongside their pivotal roles in various hepatic disease processes. Currently, most existing studies focus on single cell types or single liver diseases, lacking a systematic summary of cGAS-STING regulation under the multi-cell interaction network in the liver, this is the key research gap addressed by this review. Furthermore, drawing upon the latest preclinical research findings, this review thoroughly explores the potential and current progress of targeting the STING pathway to modulate the progression of liver diseases, thereby providing novel targets and strategies for immunotherapy in liver disorders. These findings highlight the importance of STING as a promising therapeutic target for various liver diseases, as it may be differentially expressed in various liver cells and exert complex pathogenic interactions with resident immune cells. Therefore, this review mainly focuses on the role of cGAS-STING signaling in different resident immune cells in related liver diseases.

## The cGAS-STING pathway participates in the regulation of multiple immune cells and its role in liver diseases

### Innate immune cells

#### Macrophages and the cGAS-STING pathway

Hepatic macrophages, as the core immune cells that maintain hepatic immune homeostasis and regulate disease progression, are mainly composed of tissue-resident Kupffer cells (KCs) and monocyte-derived macrophages (MoMFs). As one of the primary hepatic cell types expressing STING, they can efficiently respond to exogenous pathogen-associated molecular patterns (PAMPs) and endogenous damage-associated molecular patterns (DAMPs) through the cGAS-STING pathway [35–39], mediating a variety of biological effects including antiviral defense, anti-inflammatory or pro-inflammatory responses, and fibrosis regulation. Notably, the functional mechanisms of STING in hepatic macrophages vary considerably across disease contexts, which are closely associated with the type of STING activation (endogenous or exogenous) and clinicopathological progression. Accordingly, the bidirectional regulatory role of STING in hepatic macrophages has become a central focus in liver disease research.

Macrophage STING signaling largely determines the efficacy of innate antiviral defense during hepatitis virus infection. Hepatocytes generally exhibit low STING abundance under HBV infection, while liver macrophages with high basal STING levels are readily triggered by exogenous agonists such as DMXAA. Such stimulation elicits robust IFN-I and pro-inflammatory cytokine production, which strongly restricts HBV replication [40]. In contrast, HCV employs the NS4B protein to block the STING-TBK1 interaction, impairing downstream IFN-I productionand enabling viral immune escape [41]. In HBV-related acute-on-chronic liver failure (ACLF), massive hepatocyte injury releases large amounts of self-derived danger signals, driving persistent STING activation in macrophages to modulate cellular polarization and autophagy, and ultimately exacerbating disease progression [42].

The pathogenesis of MASLD is multifactorial and heterogeneous, characterized by sustained inflammation, hepatocyte apoptosis ,and necrosis throughout disease progression [43]. Accumulating evidence demonstrates that aberrant STING signaling contributes to MASLD and MASH progression by aggravating hepatic inflammation, lipid metabolic disturbance, hepatocyte death and insulin resistance [34]. Imbalanced secretion of pro- and anti-inflammatory mediators further sustains chronic hepatic inflammation, facilitates hepatic stellate cell (HSC) activation, and accelerates fibrotic remodeling [44, 45]. STING signaling in liver macrophages exerts a prominent dual effect on liver fibrosis, whose final outcome is governed by signaling intensity, macrophage polarization state and local microenvironment. Clinical cohort studies reveal a marked increase in CCR2^+^S100A9^+^ MoMFs, CD68^+^ KCs, and CD163^+^ macrophages in MASLD patients, with STING-positive cell proportions positively correlated with fibrosis severity [46]. In chronic metabolic liver injury, persistent accumulation of intracellular DNA and tissue damage-related molecules continuously sustains STING signaling in macrophages, driving pro-inflammatory polarization, cytokine release and HSC activation to form a vicious cycle linking metabolic inflammation and progressive fibrosis. Fibrotic progression induced by other chronic liver injuries is also closely linked to dysregulated macrophage STING cascades, highlighting the universal connection between cytoplasmic DNA sensing and hepatic fibrogenesis [47]. Sustained STING signaling further promotes IL-6 secretion and NLRP3 inflammasome activation, collectively reinforcing pro-fibrotic programs [48–51].

During the resolution phase of fibrosis, macrophages reduce extracellular matrix (ECM) secretion by clearing apoptotic hepatic stellate cells and release matrix metalloproteinases (MMPs) to degrade fibrotic tissue, thereby promoting the reversal of fibrosis [52, 53]; meanwhile, STING activation can accelerate this process through other regulatory mechanisms, mediating the body’s anti-fibrotic protective effects. Moderate STING stimulation facilitates M1 macrophage polarization, restricts HSC proliferation and alleviates liver fibrosis in experimental models [54, 55]. Meanwhile, it induces appropriate IFN-β production to block HSC activation and constrain fibrotic development [56–58]. Emerging research suggests that the activation of IRF3 and IFN-I may attenuate fibrosis by reducing the CTGF^+^VCAM1^+^ vascular endothelial cell subset and inactivating HSCs, thereby preventing liver fibrosis [59]. In adipose tissue, NRF2 deficiency alleviates systemic metabolic inflammation by restraining STING activity and promoting M2 macrophage polarization [60]. In addition, high-fat diet-induced oxidative stress activates the macrophage STING-YAP axis to regulate lipophagy and lipid droplet degradation via the TMEM205/MFN2/PDI cascade; concurrent deletion of STING and YAP markedly exaggerates hepatic lipid deposition [61], underscoring the complex functions of this pathway in metabolic‑immune crosstalk. Notably, besides macrophage-intrinsic STING function, hepatocyte STING also serves as an indispensable pathogenic mediator in metabolic liver diseases. Genomic DNA damage, gut microbial DNA translocation and lipid metabolic disorders collectively trigger cGAS-STING signaling in hepatocytes, triggering inflammation, cell apoptosis and metabolic dysfunction to facilitate MAFLD initiation and deterioration [45].

The cGAS-STING axis is widely recognized as a crucial intracellular sensing pathway in both clinical HCC specimens and experimental mouse liver models, and multiple STING-targeted agents have been validated for their anti-tumor potential. Immune-related genes profoundly affect HCC survival and carcinogenesis by remodeling the tumor immune microenvironment and regulating multiple intratumoral immune cell populations [62]. Macrophage-mediated STING signaling in HCC exhibits intricate bidirectional regulation, with its effects highly dependent on changes in the tumor microenvironment (TME) [63]. On one hand, extensive hepatocyte death releases oxidatively damaged self-DNA, which triggers persistent STING activation in tumor-infiltrating macrophages to fuel chronic inflammation, fibrotic transition and hepatocellular malignant transformation [64]. Sustained STING-IRF3-STAT1 signaling further upregulates PD-L1 expression, impairing anti-tumor immune surveillance and accelerating HCC immune escape and progression [65]. On the other hand, pharmacological or agonist-based STING stimulation drives tumor-associated macrophage (TAM) reprogramming toward the M1 anti-tumor phenotype [66]. Activated M1 macrophages further enhance T lymphocyte recruitment through the STING-IRF3 cascade, robustly boosting intratumoral T cell infiltration and enabling efficient tumor cell clearance [65, 67]. Collectively, although STING inherently initiates IFN I and inflammatory cascades, divergent upstream stimuli and microenvironmental cues redirect its downstream programs, leading to either pro-tumor or anti-tumor consequences in chronic liver diseases.

**Fig. 2:**
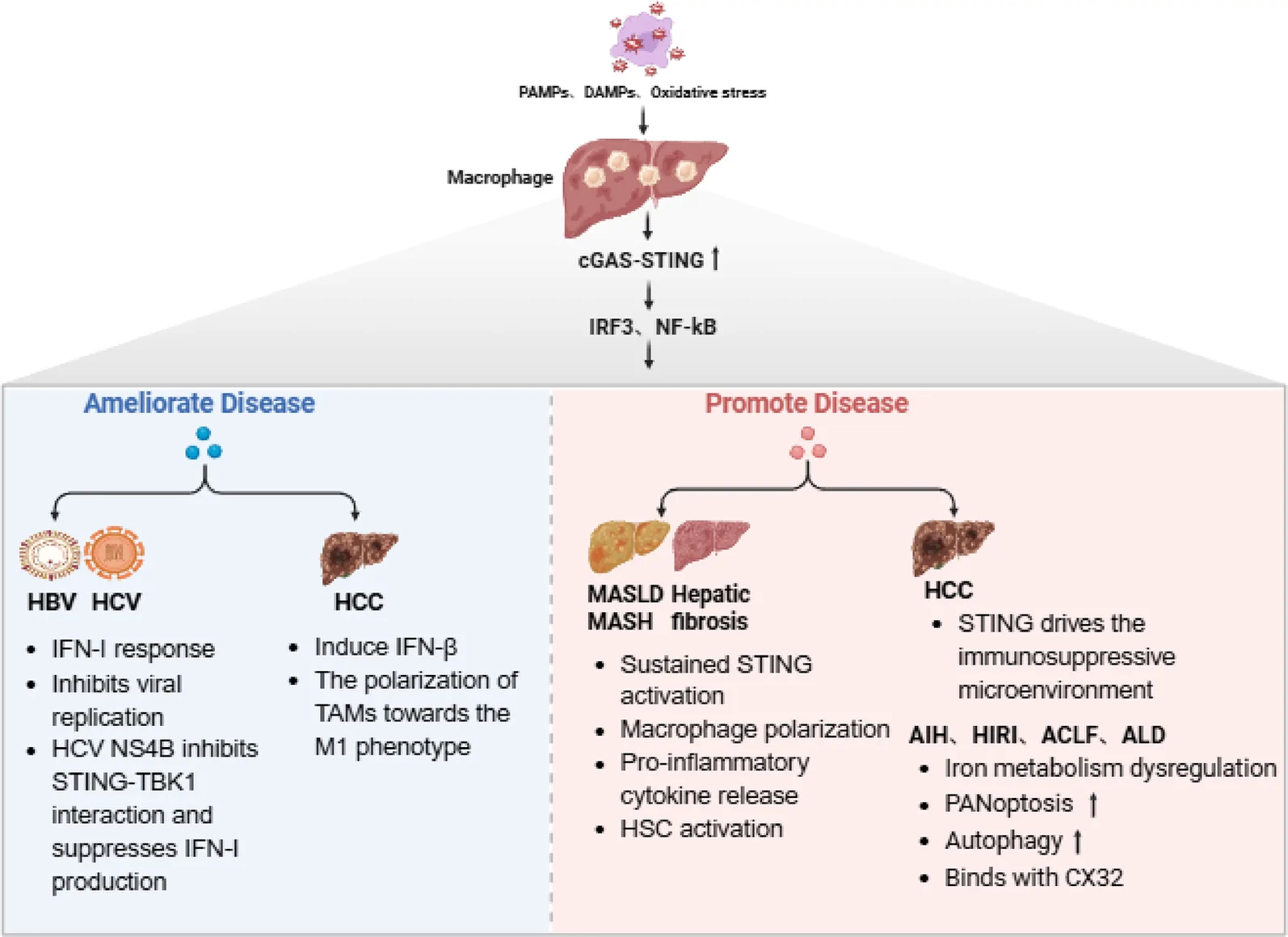
Schematic diagram illustrating the regulatory mechanisms and pathological effects of the cGAS-STING pathway in hepatic macrophages across distinct liver diseases. STING activation exerts divergent effects across different pathologies: whilst mitigating HBV and HCV infections and inhibiting HCC development, it concurrently accelerates the progression of MASLD, MASH, and liver fibrosis, thereby further promoting HCC advancement. Furthermore, STING activation accelerates the onset of AIH, HIRI, ACLF, and ALD

Beyond the above diseases, macrophage STING also participates in multiple types of acute liver injury. In hepatic ischemia-reperfusion injury (HIRI), hypoxia markedly elevates macrophage STING expression, and mtDNA leakage drives excessive STING-dependent signaling to initiate hepatocyte PANoptosis [68]. In alcoholic liver disease (ALD), chronic alcohol exposure causes mitochondrial dysfunction and endoplasmic reticulum stress, leading to the massive release of mtDNA and other damage-associated molecular patterns to activate STING signaling in hepatic macrophages. In AIH, aberrant STING activation in Kupffer cells promotes autoinflammatory cytokine secretion, and elevates iron metabolism-related proteins to trigger hepatic iron overload, oxidative stress and progressive liver injury [69, 70] (Fig. 2). In summary, hepatic macrophages including KCs and MoMFs are essential mediators of the cGAS‑STING cascade during liver pathogenesis. These cells sense external pathogenic stimuli and internal danger molecules simultaneously, thereby executing dual and context‑dependent functions. Aberrant STING activation driven by internal tissue damage sustains chronic inflammation, facilitates M2 macrophage polarization, and aggravates fibrotic and tumorous progression. In contrast, proper STING stimulation from external interventions or controlled physiological signals strengthens antiviral defense, promotes M1 phenotypic polarization, and restricts excessive fibrosis. Macrophage‑resident STING therefore acts as a vital regulatory node to maintain hepatic immune balance.

#### Dendritic cells and the cGAS-STING pathway

Dendritic cells (DCs) are indispensable antigen-presenting cells within the hepatic immune network. They determine the magnitude and direction of local immune responses. The cGAS-STING pathway acts as a core intracellular sensing axis in DCs. It recognizes abnormal cytoplasmic DNA from pathogenic invasion and tissue injury, and bridges innate immune activation with adaptive immune responses. Once triggered, DC-intrinsic cGAS-STING signaling induces the production of type I IFNs and pro-inflammatory cytokines including IL-6 and TNF-α. These biological mediators drive DC phenotypic maturation and upregulate MHC molecules and co-stimulatory markers such as CD80, CD86 and CD40. Improved antigen presentation and cross-presentation further facilitate the activation and proliferation of CD8^+^ T cells [71]. STING-activated DCs also increase CCR7 expression to enhance lymph node migration. They secrete multiple chemokines such as CXCL10 and CCL5 to recruit T cells and NK cells into inflamed or tumorous tissues [72, 73]. In addition, STING signaling fine-tunes the differentiation and functional characteristics of distinct DC subsets, including cDC1, cDC2 and plasmacytoid DCs [71, 74].

The regulatory effects of DC-associated STING are highly variable and strictly dependent on disease context. In viral hepatitis, persistent DC dysfunction weakens T cell-mediated antiviral immunity and accelerates chronic inflammatory progression [75]. Targeted activation of the cGAS-STING pathway can reverse immune paralysis during HBV and HCV infection. Bacterial-derived STING agonists such as AdrA effectively restore DC immune activity. They promote the secretion of IFN-Ⅰ and TNF-α, and ultimately reduce hepatic viral load and peripheral viremia [76]. Similarly, human-specific STING agonists strongly facilitate DC maturation and antigen presentation to strengthen viral clearance [77].

In HCC, DC cGAS-STING activation is widely regarded as a central anti-tumor mechanism. Appropriate STING stimulation remodels the tumor immune microenvironment, promotes intratumoral infiltration of cytotoxic CD8^+^ T cells and NK cells, and restrains tumor malignant progression [78]. Nevertheless, DC-STING signaling exhibits obvious dual properties under chronic pathological conditions. Sustained STING activation can drive DCs to express high levels of PD-L1. This process promotes Treg accumulation and induces local immune tolerance, thereby mediating resistance to immune checkpoint therapy in HCC [79]. In chronic inflammatory liver diseases such as MASH and autoimmune hepatitis, continuous STING-driven inflammatory factor secretion by DCs amplifies hepatic inflammation and exacerbates tissue damage [80, 81]. Of note, the detailed mechanisms of DC-STING signaling in autoimmune liver disorders remain poorly defined, representing an important research gap in this field.

Beyond cell-autonomous STING activation in DCs, STING signaling in other hepatic cell populations can indirectly modulate DC biological functions. In MASH-related fibrosis, NK cell STING activation induced by insulin elevates IFN-γ secretion to promote DC maturation and antigen presentation [82]. In radiation-induced liver injury, STING activation in Kupffer cells triggers the release of IL-1β and IL-18. These pro-inflammatory cytokines further drive DC recruitment and overactivation to aggravate tissue inflammation [83].

Multiple exogenous STING regulators and chemical drugs modulate liver disease progression by targeting DCs. The bacterial cyclic dinucleotide C-di-AMP initiates DC STING activation to boost anti-HCC immunity and radiosensitivity in a cGAS-independent manner [84]. Triptolide drives aberrant STING signaling and endoplasmic reticulum stress to aggravate liver injury. STING blockade restrains cDC and invariant natural killer T (iNKT) cell activation, thereby alleviating triptolide-induced hepatic damage [85]. In HCC, STAT3 silencing strengthens sorafenib-mediated tumor apoptosis. Tumor cell-derived DNA fragments trigger cGAS-STING signaling in CD103^+^ DCs. Subsequent type I IFN production potentiates the anti-tumor activities of CD8^+^ T cells and NK cells [78]. In addition, CD47 blockade elevates tumor DNA uptake by CD103^+^ DCs, amplifying NK cell STING activation to suppress HCC progression [86].

Collectively, the STING pathway achieves comprehensive immune regulation through direct and indirect modulation of DCs. Most current studies focus on STING activation in hepatic non-parenchymal cells. The biological function of the cGAS-STING pathway in hepatocytes remains underappreciated, even though low hepatic STING expression exerts decisive impacts on HBV-related liver pathogenesis.

#### Natural killer cells and the cGAS-STING pathway

Natural killer cells (NK cells), serving as the core cytotoxic lymphocytes within the liver’s innate immune system, exhibit significantly higher abundance in the liver than in peripheral blood. This regional enrichment enables them to rapidly detect local pathogen invasion and cellular abnormalities within the liver [87]. Their functional activity relies on the dynamic equilibrium between surface inhibitory receptors (such as the KIR family) and activating receptors (such as NKG2D), enabling precise recognition and elimination of virus-infected cells and tumor cells. Concurrently, they secrete effector molecules including IFN-γ and TNF-α, playing an irreplaceable role in hepatic immune surveillance, regulation of inflammatory homeostasis, and tissue repair [88, 89].

Recent studies have confirmed that the cGAS-STING pathway constitutes a crucial molecular mechanism regulating NK cell function. It influences NK cell activity through direct and indirect regulatory modes to shape the progression and outcome of liver diseases. Regarding direct regulation, activation of the STING pathway in NK cells primarily follows the classical TBK1-IRF3 pathway, ultimately initiating transcription of effector molecules such as IFN-γ, perforin, and granzyme B [90]. This sustainably boosts NK cell functionality. For instance, in HCC, direct activation of the cGAS-STING pathway maintains the stability of the TCF-1^+^ NK cell population (possessing enhanced sustained IFN-γ secretion capacity and anti-exhaustion properties), effectively countering immunosuppressive pressures within the HCC microenvironment [90]. In colorectal cancer liver metastases, this mechanism sustains NK cell infiltration and cytotoxicity [23]. Conversely, STING deficiency markedly reduces NK cell IFN-γ expression and degranulation capacity, directly demonstrating STING’s essential role in NK cell function [90]. For instance, in HBeAg-negative CHB patients, STING expression levels are markedly reduced in NK cells, with STING levels positively correlated with NK cell degranulation capacity. The possible mechanism is that HBsAg inhibits STING expression in NK cells through STAT3, impairing their response to cGAMP [91].

The indirect regulatory mechanism is primarily manifested in certain antigen-presenting cells (such as DCs and macrophages) or tumor/infected cells, where cGAS-STING pathway activation indirectly modulates NK cell infiltration, activation, and proliferation through cytokine release. In DC-mediated indirect regulation, STING activation in CD103⁺ DCs3^+^ leads to CXCL9 secretion, which chemotactically promotes NK cell infiltration into liver injury sites or tumor sites. Concurrently, DC-secreted IL-12 activates NK cells to secrete IFN-γ [86]. In macrophage-mediated indirect regulation, cGAS-STING activation triggers IL-18/IL-1β production and upregulates 4-1BBL expression. The synergy between cytokine signaling and 4-1BBL/4-1BB co-stimulation significantly enhances NK cell cytotoxicity [23]. In B cell-mediated indirect regulation, cGAMP-activated B cells secrete IL-35, which simultaneously suppresses tumor and NK cell proliferation, resulting in a contradictory effect of ‘tumor suppression versus immune suppression [92]. This multi-cell indirect regulatory model underscores the cGAS-STING pathway’s pivotal role in the hepatic immune network and provides a mechanistic basis for understanding the NK cell functional microenvironment.

#### Natural killer T cells and the cGAS-STING pathway

The liver is the organ with the highest concentration of natural killer T cells (NKT cells) in the body, accounting for 20–35% of hepatic lymphocytes in mice and 10–15% in humans [93]. Based on TCR diversity and functional differences, NKT cells comprise two core subtypes with markedly opposing roles: type I NKT cells, expressing only invariant TCRs, rapidly secrete large amounts of cytokines (e.g., IFN-γ and IL-4) upon stimulation, bridging innate and adaptive immunity to participate in anti-infective and antitumor responses or exacerbate inflammatory damage [94]; type II NKT cells express diverse TCRs and primarily exert immunosuppressive functions, promoting tumor progression and metastasis [95–98]. Concurrently, NKT cells regulate DC maturation, B-cell class-switching, and the activation of CD8^+^ T or NK cells [99–103], rendering them pivotal “hub cells” linking the innate and adaptive immune systems in the liver, as well as crucial “effectors” mediating the cGAS-STING pathway’s influence on liver disease progression.

Although no direct evidence currently exists demonstrating STING expression on NKT cells, studies confirm that STING pathway activation in other immune cells can indirectly regulate NKT cell function via multi-layered cellular crosstalk networks. It is worth noting that the cGAS-STING pathway exhibits marked liver disease context dependency in NKT cell-mediated responses. In acute or chronic liver inflammation (e.g., drug-induced liver injury and AIH), STING pathway activation in other immune cells promotes the release of pro-inflammatory factors (e.g., IFN-γ and osteopontin (OPN)) by type I NKT cells), exacerbating hepatocyte necrosis and autoimmune responses [104, 105]. However, in HCC, the STING pathway not only exerts the antitumor activity of type I NKT cells via APCs, but also promotes immunosuppression through type II NKT cells—such as by recruiting regulatory T cells and secreting Th2 cytokines—which indirectly facilitates tumor progression [106, 107]. Furthermore, activated NKT cells can suppress Th1 differentiation by eliminating the IL-12^+^DC1 subset, forming a bidirectional regulation-feedback network that maintains hepatic immune homeostasis [108]. In MASLD, KC-STING activation releases IL-6 and TNF-α, enhancing NKT and CD8^+^ T cell infiltration and promoting fatty liver inflammation progression [109].

In oncology, STING agonist-antibody conjugates (ADCs) increase the proportion of activated NKT cells in tumors and draining lymph nodes [110]; cell cycle checkpoint kinase 1 inhibitors activate DCs via the tumor cell cGAS-STING pathway, driving CD1d-dependent IFN-γ secretion by NKT cells to synergise antitumor effects [111]; systemic blockade of CD39/ENPP1 also recruits NKT cells via DC or macrophage STING signaling to enhance immune responses [111]. Although direct evidence regarding the indirect regulation of NKT cells by STING in liver tumors remains lacking, the mechanistic characteristics of STING agonist-antibody conjugates and cell cycle checkpoint kinase 1 inhibitors described above may provide valuable insights for further exploration.

In summary, current research indicates that the STING signaling pathway primarily regulates NKT cell-mediated immune responses in various liver diseases through indirect and context-dependent mechanisms. The specific molecular mechanisms underlying its regulatory role in liver pathology remain to be further explored.

#### Neutrophils and the cGAS-STING pathway

Neutrophils, serving as the “first-responder effector cells” in hepatic innate immunity, possess robust immunoprotective capabilities and dynamic regulatory properties. Although resident neutrophils in the liver are scarce, they can be extensively recruited to hepatic sinusoids during acute injury, infection, or chronic inflammation. Thereby they exert dual regulatory effects on disease outcomes: early on, they maintain hepatic homeostasis by eliminating pathogens and activating adaptive immunity; later, they may drive liver fibrosis by secreting pro-inflammatory factors such as IL-1β and TNF-α, or induce macrophages to switch to a reparative phenotype [112–116].

Neutrophils themselves exhibit extremely low, often undetectable levels of STING expression. Forced expression and activation of STING in neutrophils induces severe pathological consequences, including weight loss, reduced survival rates, and alopecia in mice, accompanied by systemic autoinflammatory features such as splenomegaly, thymic atrophy, and widespread lymphadenopathy, alongside markedly elevated serum levels of multiple cytokines and chemokines [117]. This suggests that low STING expression in neutrophils may represent a self-protective mechanism during differentiation and survival, preventing self-damage from excessive activation.

Presently, only one study supports direct regulation of neutrophil function by their intrinsic STING: in pulmonary ischaemia-reperfusion injury, mtDNA leakage within neutrophils activates the endogenous cGAS-STING pathway, promoting NET formation and driving IL-1β/CXCL10-mediated inflammatory cascades that exacerbate lung injury [118]. Although this study focused on lung injury, the concurrent observation of mtDNA release and NET accumulation in neutrophils during hepatic ischaemia-reperfusion suggests that liver neutrophils may similarly contribute to injury via direct STING activation. However, given the low endogenous expression of STING in neutrophils, its impact is likely weaker than that of indirect regulatory pathways.

In liver diseases, the cGAS-STING pathway primarily regulates neutrophil function through indirect mechanisms. In oncology, IFN-I released upon activation of the tumor cell-intrinsic cGAS-STING pathway induces an increased proportion of the Ly6Ehi neutrophil subpopulation within the hepatic microenvironment. This significantly enhances tumor sensitivity to PD-1 inhibitors, eliciting therapeutic responses in previously non-responsive tumors [119]. In MASLD, activation of the cGAS-STING pathway in other immune cells induces the release of chemokines such as CXCL1 and CXCL10, recruiting Ly6Ehi neutrophils to infiltrate fatty liver regions, thereby exacerbating hepatocyte lipotoxicity and inflammatory responses [119]. In acute inflammation such as sepsis, or hepatic ischaemia-reperfusion injury (HIRI), activation of the cGAS-STING pathway in other immune cells (e.g., macrophages, endothelial cells) can exacerbate neutrophil NET formation (NETosis) via the IRE1α-ASK1-JNK signaling pathway, whilst excessive NET accumulation induces hepatic sinusoidal microthrombosis and hepatic cell ischaemia-hypoxia injury [120]. This finding expands our understanding of neutrophil involvement in acute inflammatory mechanisms.STING signaling controls neutrophil recruitment and functional activity mainly through indirect pathways. Such regulation varies greatly across distinct liver diseases in a context‑dependent manner.

#### Eosinophils, mast cells, and basophils in the cGAS-STING pathway

Eosinophils, mast cells, and basophils constitute multifunctional cell subsets within the innate immune system, possessing dual roles in both “inflammatory regulation” and “tissue repair”. All three cell types highly express FcεRI, which, upon IgE-allergen binding, triggers the release of histamine, leukotrienes, and cytokines, thereby modulating the intensity and duration of inflammatory responses [121–124]. Under physiological conditions, none of these cell types are resident in the liver; they are primarily recruited to sites of liver injury or infection via chemotactic signals from other immune cells, thereby influencing disease progression. Although substantial STING gene expression has been detected in all three cell types, the association between their intrinsic STING activity and functional changes, along with their precise mechanisms in the immune regulation of liver diseases, remains unexplored. Currently, indirect evidence linking these cells to the cGAS-STING pathway is also scarce.

### Adaptive immune cells

#### T lymphocytes and the cGAS-STING pathway

T cells originate from haematopoietic stem cells in the bone marrow. Following selection within the thymus, they differentiate into mature populations with MHC restriction. These primarily comprise CD4^+^ helper T cells (which regulate immune response magnitude by secreting cytokines) and CD8^+^ cytotoxic T cells (which directly eliminate virus-infected or tumor cells via the perforin/granzyme system) [125, 126]. Their activation relies on antigen-presenting cells (such as DCs) presenting antigen peptides via MHC-I/II molecules, forming a dual activation mode involving antigenic and co-stimulatory signals.

T cells are the core effectors of adaptive immunity in the liver. Notably, the expression levels of the STING protein in T cells are comparable to those in macrophages [126], which provides a structural foundation for endogenous STING to directly regulate T cell function. It has been reported that during thymic development of T cells, STING expression is elevated at the double-negative (DN) stage, decreases significantly at the double-positive (DP) stage, and rises again at the single-positive (SP) stage. Forced activation of STING signaling during the DN stage hinders T cell development and promotes a shift towards the γδ T cell lineage, indicating that abnormal STING signaling can disrupt normal T cell differentiation [117]. In mature T cells, endogenous STING activation not only induces IFN-I production and ISG expression, similar to its role in innate immune cells-but also triggers cellular stress and death pathways, though with notable differences between in vitro and in vivo settings [126]. This may suggests that T cell-intrinsic STING is involved in both immune activation and cell survival regulation, although the underlying mechanisms remain unclear.

**Fig. 3:**
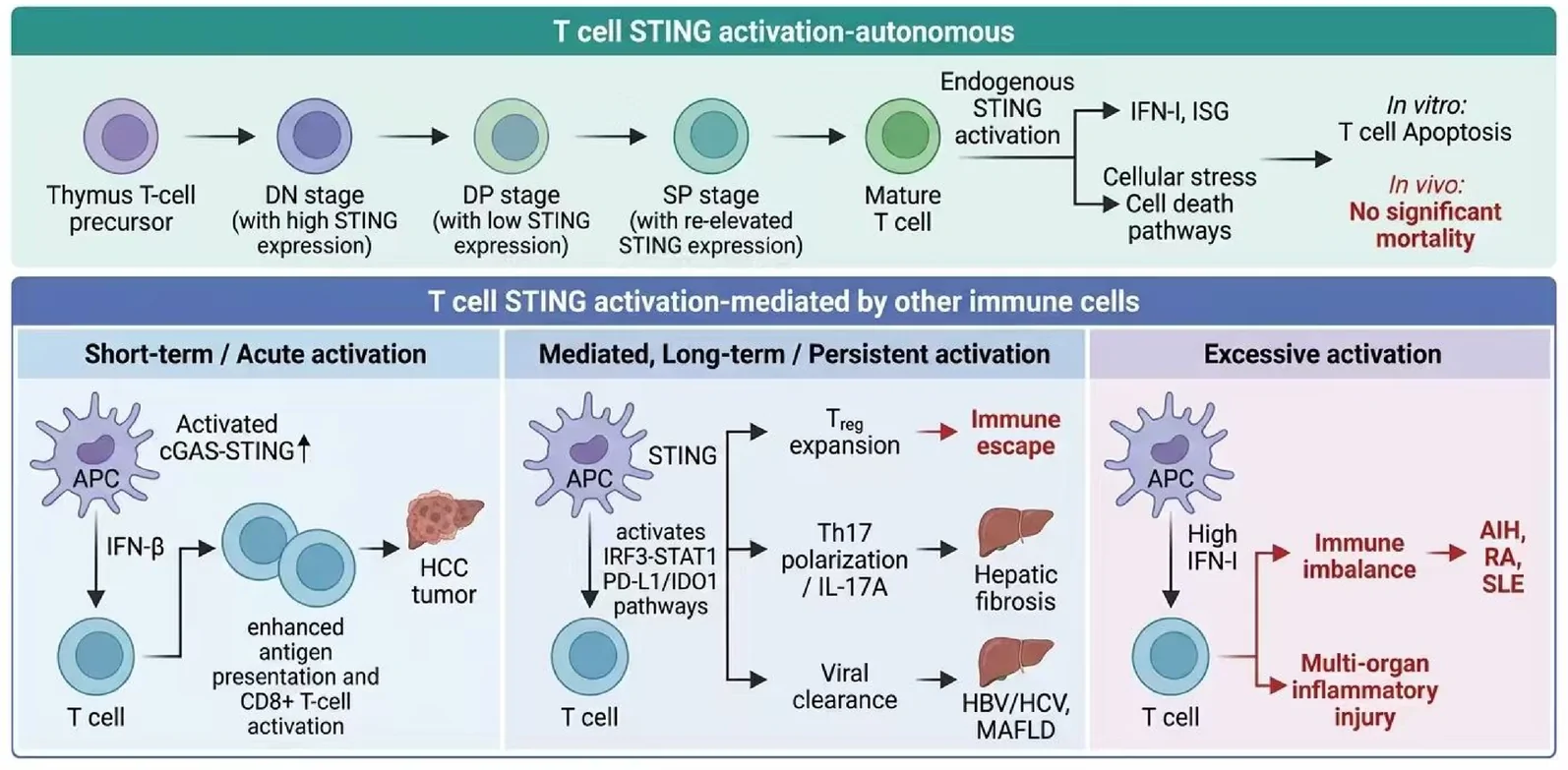
Dual regulatory mode and pathological effects of the cGAS-STING pathway on T cells. Direct regulation: High STING expression in thymic T cells during the DN stage; abnormal activation at this stage impedes their development and shifts towards γδ T cells; Activation of endogenous pathways in mature CD4^+^/CD8^+^ T cells induces IFN-I and ISG expression while regulating cellular stress and death; Antigen-presenting cell (APC, primarily DCs)-mediated indirect regulation: Short-term activation enhances DC antigen presentation efficiency, promoting CD8^+^ T cell activation to suppress HCC progression or reverse HBV/HCV infection-associated T cell exhaustion; Prolonged/excessive activation, however, induces Treg expansion and CD4^+^ T cell Th17 polarization via the IRF3-STAT1 axis, exacerbating HCC immune evasion, MAFLD inflammation, liver fibrosis, and AIH progression. Furthermore, dysregulated pathway activation can drive T cell dysregulation and multi-organ damage in autoimmune diseases such as RA and systemic lupus erythematosus (SLE) through sustained IFN-I induction

The cGAS-STING pathway also regulates T cell function through an indirect, APC-mediated mode, which represents the current mainstream research direction. This mechanism primarily shapes T cell responses by activating the STING pathway within APCs. For instance, within the HCC microenvironment, DNA damage in tumor cells triggers STING signaling activation within DCs. This process facilitates the infiltration of CD8^+^ T cells into tumor sites and enhances their cytotoxic activity [127]. However, the effects of this indirect T cell regulation exhibit activation-timing dependency: short-term acute activation enhances CD8^+^ T cell cytotoxicity via DC-mediated indirect regulation; whereas sustained STING activation upregulates PD-L1 and IDO1 expression through the IRF3-STAT1 axis, not only inducing Treg expansion but also directly suppressing CD8^+^ T cell function, ultimately establishing an immune-evasive microenvironment [65]. A similar pattern is observed in MASLD: early STING activation in DCs promotes CD8^+^ T cell activation to maintain immune surveillance and delay carcinogenesis; however, persistent STING activation under chronic inflammatory conditions drives CD4^+^ T cells towards Th17 subtype polarization, exacerbating the vicious cycle of inflammation and fibrosis through IL-17 A secretion [128]. Another form of prolonged activation is excessive stimulation, primarily observed in AIH. Multiple studies indicate that the STING pathway in AIH can be abnormally activated by self-DNA or mitochondrial DNA, promoting the release of IFN-I and inflammatory cytokines, thereby excessively activating adaptive immune responses such as T cell responses [129]. In certain autoimmune diseases, this abnormal T cell activation may also cause multi-organ inflammatory damage [130]. However, in chronic viral infections (e.g., HBV, HCV) that induce T cell exhaustion-related liver diseases, STING activation can reverse T cell exhaustion by enhancing antigen presentation by DCs, thereby facilitating viral clearance [131] (Fig. 3).

#### B lymphocytes and the cGAS-STING pathway

B cells, as the core effectors of hepatic humoral immunity, constitute nearly 6% of the liver’s total cellular population. In mouse livers, B cells may constitute up to 50% of hepatic lymphocytes [132]. This high enrichment enables them to rapidly respond to local pathogenic invasion and injury signals within the liver, participating in various hepatic disease processes by producing antibodies, assisting T cell activation, secreting inflammatory cytokines, and participating in antiviral immunity [133, 134].

Current research has established that the direct regulation of B cell function by the cGAS-STING pathway is intrinsically linked to its receptor (BCR). Activation of the STING pathway in B cells significantly upregulates the expression of the co-stimulatory molecule CD86 on their surface. This synergises with BCR signaling, further enhancing B cell activation levels [135]. However, under physiological conditions, B cell-specific STING knockout also markedly increases BCR expression and signaling, suggesting a potential self-balancing or negative feedback mechanism to maintain immune homeostasis [136].

To date, there is no definitive evidence demonstrating that intrinsic or extrinsic STING activation in B cells contributes to the pathological progression of liver diseases. Although STING activation in B cells has been verified to promote autoantibody production in systemic lupus erythematosus (SLE), its regulatory role in autoimmune hepatitis (AIH) remains unreported, and relevant evidence in other hepatic disorders is still quite limited. By reviewing relevant studies on other diseases, we found that the cGAS-STING pathway exhibits pronounced bidirectional effects on B cell regulation, with functional outcomes determined by activation intensity, cell type, and microenvironmental signals. In immune defense, STING activation in non-hematopoietic cells (e.g., mouse embryonic fibroblasts, melanoma cells, and HCC cells) mainly induces IFN-I secretion, thereby supporting antiviral and antitumor responses; whereas in malignant B cells, STING activation primarily triggers apoptosis to eradicate abnormally proliferating B cells [137]. Mechanistically, prolonged STING activation—due to impaired degradation—initiates apoptosis [137], and STING also interacts with Poly ADP-ribose (PAR) to modulate B cell survival and activation [138]. Conversely, in promoting immune evasion, STING activation induces B cells to secrete IL-35 via IRF3 signaling (independent of IFN-I), significantly suppressing NK cell proliferation and IFN-γ secretion. This creates an immunosuppressive microenvironment contributing to tumor immune escape [92, 139]. The relationship between B cell STING and liver diseases remains to be further explored, and the above findings from extrahepatic disease studies provide a basis and reference for subsequent investigations.

## The cGAS-STING signaling pathway as a potential therapeutic target for liver diseases

In recent years, research on drugs and regulatory mediators targeting the cGAS‑STING pathway has expanded rapidly. In cardiovascular diseases [140], moderate activation of STING can induce an effective immune response against myocarditis caused by myocarditis virus. In malignancies such as gastric cancer [141], XRCC1 deletion drives STING pathway activation to facilitate tumor immune evasion. These findings further validate the great potential of STING as a novel therapeutic target. Common risk factors for liver diseases include lipid homeostasis disorders, bile acid overload, oxidative stress ,and persistent inflammation, which serves as the most critical contributor. The functional heterogeneity of STING is predominantly determined by cell types. Distinct innate and adaptive immune cell subsets in the liver display divergent downstream signaling profiles and thus trigger completely opposite pathological outcomes. Both excessive activation and functional deficiency of STING disrupt the hepatic immune microenvironment, induce cellular dysfunction and aggravate liver disease progression. Therefore, given the diversity of immune cells in the liver and the complex, context-dependent role of STING in liver diseases, we cannot simply conclude that STING activation invariably promotes inflammation; instead, we must comprehensively consider the local microenvironment and the stage of disease progression. This fundamental difference leads to inconsistent and even contradictory therapeutic outcomes when targeting the cGAS‑STING pathway across various liver disorders. Therefore, it has become an urgent research bottleneck to achieve precise pathway regulation, abandon generalized intervention strategies, and maximize clinical therapeutic benefits.

### Traditional STING regulation strategies

#### STING agonists (Table 1)

Currently, STING agonists are advancing rapidly in tumor immunotherapy, with a primary focus on solid tumors. However, most candidates remain in Phase I clinical development, requiring further optimization to balance efficacy and toxicity. Early-generation STING activators were limited by structural and stability deficiencies, rendering them unsuitable for systemic administration, and preliminary clinical results for STING modulators have been highly inconsistent.

In recent years, novel cyclic dinucleotide (CDN)-based and non-CDN-based STING agonists have been developed, exhibiting significantly improved potency and binding affinity, as well as the potential for systemic delivery [13]. Low to moderate doses of CDNs and their derivatives, such as 3’3’-cAIMP and cGAMP, enhance crosstalk between hepatocytes and immune cells in the liver by activating STING, and trigger immune responses via the induction of IFNs, interferon-stimulated genes (ISGs), and chemokines, thereby exerting antiviral and antitumor effects [67, 142–144]. In contrast, non-CDN small molecules such as DMXAA and CMA are capable of activating mouse STING (mSTING). Although DMXAA can inhibit HBV replication, high doses also activate hepatic macrophages and induce hepatic steatosis and inflammation [40, 145–147]. Regrettably, DMXAA, an anti-tumor agent acting as a vasculotoxic agent, failed to activate human STING (hSTING) in clinical trials. However, ongoing research focuses on synthesising hSTING-targeted derivatives, with the mildly potent hSTING agonist α-mangostin demonstrating promising potential [148]. It may specifically address the species-specific limitations of traditional agonists such as DMXAA, which are unable to activate hSTING, thereby filling a gap in the development of hSTING-targeted agonists.Beyond classical small-molecule agonists, the peptide drug Gv1001 and the chemotherapeutic agent doxorubicin activate the STING signaling pathway by inducing mitochondrial stress and generating dsDNA, thereby inhibiting HBV replication [40, 149, 150].

Given the unique advantages demonstrated by STING agonists in enhancing antiviral efficacy, another more established application lies in their use as vaccine adjuvants-a research direction that was also among the earliest to undergo clinical translation. cGAMP, as a potent STING ligand, significantly amplifies the immune response intensity of HBV vaccines [151]. Compared to TLR agonists, STING agonists not only induce stronger antiviral responses but also mitigate inflammatory tissue damage by reducing pro-inflammatory cytokine secretion, thereby offering superior safety profiles [40]. STING agonists such as cGAMP and c-di-AMP have been validated as candidate adjuvants for HBV/HCV vaccine development, enhancing adaptive immune responses through synergistic activation of innate immunity [144]. These significant findings not only validate the potent efficacy of STING pathway agonists in enhancing vaccine immune responses but also highlight their broad application prospects and translational potential as novel vaccine adjuvants in antiviral vaccine design.

The primary challenges with current STING agonists include low monotherapy response rates (below 20%), poor stability, and systemic administration readily inducing cytokine storms [152–155]. To date, no STING agonist has advanced to Phase II clinical trials with satisfactory efficacy, primarily due to the need to balance therapeutic immune activation with systemic toxicity, presenting significant challenges for researchers. Another challenge is the different effects of STING agonist in different diseases. Recent studies have reported that the bidirectional roles of STING in the same disease or at different disease stages can be attributed to three major factors: (i) cell type dependency; (ii) treatment timing and duration; (iii) biased signaling transduction [156]. However, the specific regulatory mechanisms of STING at different progressive stages of liver diseases such as HBV infection, MASLD and HCC remain unclear. Therefore, in the future, the application of STING agonists in disease treatment should strictly control the dosage, administration route, and treatment timing, and closely monitor inflammatory parameters.

#### STING inhibitors (Table 2)

Abnormal activation of the STING pathway constitutes a key mechanism in certain autoimmune diseases, exhibiting close associations with Aicardi-Goutières syndrome (AGS), SLE, and autoimmune liver disease. Synthetic small-molecule inhibitors primarily block pathway activation in preclinical models by inhibiting STING dimerization or competitively occupying the cGAMP binding site [157, 158]. The newly discovered UM-200 inhibitor effectively blocks STING-dependent signaling in both mice and humans, suggesting that it holds promise as a potential effective antagonist for the treatment of STING-associated inflammatory and autoimmune diseases [159]. The selective STING inhibitor ISD017 specifically suppresses ISG expression in SLE patients [160], while cGAS small-molecule inhibitors block abnormal STING activation in MASH [161]. Furthermore, the selective STING inhibitor C-176 alleviates lipid peroxidation and ferroptosis in AML12 cells [162], whilst H-151 mitigates hepatic ischaemia-reperfusion injury by reducing mitochondrial damage in hepatocytes and inhibiting STING activation in macrophages [163]. Certain derivatives of natural products, such as the plant-derived lactone Obtusilactone B, the leguminous compound Brasilin, and the tea polyphenol EGCG (epigallocatechin gallate), exert inhibitory effects primarily via indirect regulation [153]. It is worth noting that pentagalloylglucose (PGG), a natural compound, alleviates acetaminophen-induced acute liver injury by modulating inflammation mediated through the cGAS-STING pathway [164]. Moreover, traditional Chinese herbal compound preparations demonstrate unique value in improving liver diseases. Examples include the formulas Ertao Tang and Linggui Zhugan Tang, alongside ginsenoside Rd, which improve hepatic lipid deposition, fibrosis, and acute liver injury by multi-target inhibition of the STING pathway [165–168], while total sesquiterpenoids from Salvia miltiorrhiza have been demonstrated to play a significant role in alleviating inflammation and autoimmune diseases [169].

#### Repurposing of existing drugs with STING-modulating activity

Certain clinically available medications, though not specifically developed for the STING pathway, have been found through research to exert therapeutic effects by influencing this pathway. They may be categorised accordingly based on their mechanisms of action. Both 3,5-diiodo-L-thyroxine and 3,5,3’-triiodo-L-thyroxine are linked to aberrant cGAS-STING signaling and multiple sterile inflammatory disorders including non-alcoholic fatty liver disease and obesity. Impaired mitochondrial quality control and defective autophagy are central to the related pathogenesis. These two iodothyronines restore oxidative balance by boosting antioxidant activity, activating mtDNA repair pathways (OGG1, APE1, POLγ), limiting mtDNA damage, and enhancing autophagy [170]. Furthermore, although some commonly used clinical drugs were not specifically developed to target the STING pathway, they can exert therapeutic effects by regulating this pathway. For example, low-dose sorafenib combined with STAT3 inhibition can directly eliminate tumor cells and improve the tumor microenvironment [78], while remdesivir can inhibit related signaling pathways, thereby alleviating high-fat-induced inflammation and lipid metabolism abnormalities [171]. This suggests that the STING pathway serves as a key node in regulating various pathological processes, providing a direction for the repurposing of existing drugs while laying the foundation for new therapeutic strategies for inflammatory diseases, thereby facilitating the translation of basic research into clinical practice.

### Novel strategies facilitating STING drug development

#### Targeted delivery (agonists, inhibitors)

In recent years, novel regulatory strategies for the STING pathway have continually overcome traditional limitations. Among these, targeted delivery systems—which can precisely target lesions and reduce systemic toxicity—have become a key focus of research and development, while the application of antibody-drug conjugates (ADCs), exosome platforms, and nanotechnology platforms has further expanded the scope of exploration in this field.

Antibody-drug conjugates (ADCs) have demonstrated significant potential in preclinical studies; STING ADCs have shown far superior efficacy in inducing immune responses and tumor cell death compared to free STING agonists [172]. Although the first STING ADC to enter clinical trials (XMT-2056) was discontinued in 2023 due to concerns over adverse reactions, it provided valuable insights for subsequent research and development; Exosome-based delivery platforms also possess unique advantages. Exosome-delivered STING modulators, utilizing engineered surface glycoproteins, can efficiently achieve intratumoral targeted delivery, induce potent tumor-specific immune responses, and significantly reduce systemic cytokine levels, outperforming free drugs [173]; nanotechnology platforms have greatly expanded the application boundaries of STING pathway regulation, PC7A nanoparticles can enhance antigen delivery and specific immune activation through spatiotemporal coordination mechanisms; liposomal nanoparticles demonstrate outstanding performance in cGAMP delivery; and ONM-500, as a novel STING-activating nanovaccine, can effectively deliver antigens to lymph nodes, all of which have shown good antitumor activity in animal models [174, 175].

Precision regulatory strategies for liver diseases are also emerging. PLA nanoparticles—cationic lipid complexes (DP-D) can co-load the STING agonists DMXAA and DDAB, continuously activating the cGAS-STING pathway in a chronic hepatitis B (CHB) model to enhance antiviral immunity [147]. human umbilical cord mesenchymal stem cell exosomes (Huc-MSCs-exo) delivering miR-24-3p can inhibit STING activation in macrophages, promote the polarization of hepatic macrophages toward the M2 phenotype, and thereby improve liver fibrosis [176]. Nanotechnology platforms have further expanded delivery capabilities: Fe/Mn nanovaccines indirectly activate intracellular pathways in DCs by inducing pyroptosis in tumor cells to release DAMPs, thereby reversing immune tolerance [177–179]; the RCM-Lip nanoplatform simultaneously induces tumor apoptosis and delivers the agonist MSA-2 to DCs under ultrasound-controlled release, promoting systemic antitumor immunity [180]; while pH-sensitive nanoparticles achieve specific STING activation in DCs, substantially reducing systemic inflammatory risks and establishing a novel paradigm for hepatocellular carcinoma immunotherapy [181].

Regarding immunotoxicity regulation, microparticle-delivered STING agonists indirectly activate NK cells via APC-mediated IFN-I release, demonstrating markedly superior killing capacity enhancement compared to soluble cGAMP’s limited effect of merely inducing surface marker upregulation [182]; Zhang Feng’s team utilised albumin-manganese nanoparticles to deliver Mn^2+^ to hepatic stellate cells, activating cGAS-STING to induce senescence and enhance NK cell clearance capacity, thereby successfully reversing liver fibrosis [183]. Genetic knockout of SCAP or inhibition of STING alleviates hepatic steatosis and inflammation, suggesting that targeting the SCAP-STING axis may offer a new therapeutic strategy for Lean MASLD [184]. Furthermore, SYNB1891 (an engineered strain of non-pathogenic E. coli) based on a bacterial vector can localize to the tumor microenvironment to produce cyclic dinucleotides, inducing INF-I production and tumor regression; its safety and efficacy for intratumoral administration are currently being evaluated in a Phase I clinical trial [185]. The application of these emerging technologies not only enhances the biological effects of STING-targeted drugs in precisely intervening at the cellular level but also significantly reduces the risk of systemic adverse reactions.

In our view, regardless of the carrier or delivery strategy employed, it may be challenging to completely avoid uptake by non-target cells during targeted delivery. In the metabolically active liver, non-specific uptake of carriers by hepatocytes and non-target immune cells can lead to abnormal activation of the STING pathway, triggering local or even systemic inflammatory responses. Furthermore, significant species differences severely limit clinical translation. While STING activation mediated by certain nanocarriers demonstrates significant antitumor effects in mouse models, differences in STING protein structure, expression patterns, and downstream signaling between humans and mice make it difficult to replicate these effects in preclinical human studies. Furthermore, if the delivery system cannot be controlled to degrade, sustained STING activation may disrupt immune homeostasis, leading to immune cell exhaustion or excessive inflammation. These are all issues that must be considered in subsequent drug development.

#### Combination therapy

Combination therapy significantly enhances the anti-tumor efficacy of immune cells induced by the cGAS-STING pathway through multi-target synergistic effects. Within the immune checkpoint blockade domain, the combination of cGAMP and PD-L1 antibodies elicits potent antitumor responses in HCC [186]. Radiofrequency ablation (RFA) combined with DNA therapy synergistically activates the DCs-CD8^+^ T cell immune axis by restoring tumor antigen presentation capacity. This approach doubled postoperative survival in mouse models and markedly suppressed tumor recurrence, while the combination of STING agonists and PD-1 inhibitors overcomes the limitations of monotherapy by synergistically amplifying tumor-infiltrating lymphocytes (TILs) and reversing their exhausted phenotype [187, 188]. Within chemotherapy synergistic strategies, the combination of teniposide and hyperbaric oxygen amplifies IFN-I and NF-κB signaling pathways, enhancing DC maturation and thereby significantly boosting the therapeutic efficacy of PD-1 antibodies [189, 190]. In addition, STING-related tumor vaccines represent another emerging platform with great promise. For example, STINGVAX is a vaccine that combines GCSF-expressing cells with synthetic CDNs (as adjuvants); subcutaneous administration of STINGVAX induces STING-dependent IFN-I and IRF3 expression and generates an antitumor response, particularly when used in combination with PD-1 blockade therapy [191]. These studies suggest that multi-strategy combinations targeting the cGAS-STING pathway—such as immune blockade and ablation therapies—represent a crucial direction for overcoming monotherapy limitations and enhancing antitumor efficacy, while also informing clinical protocol design.

#### Microbiome therapy

Microbiome interventions demonstrate therapeutic potential through differential modulation of the STING pathway. Lactobacillus rhamnosus GG (LGG) activates STING signaling in DCs, promoting CXCL10 secretion and enhancing CD8^+^ T cell tumor infiltration to strengthen antitumor immunity [192]. Mycobacterium paragordonae, via its metabolite c-di-GMP, activates the DCs-STING-IFN-I axis to drive broad-spectrum antitumor immunity in NK cells [193]. In contrast, Bifidobacterium triple live powder significantly reduces pro-inflammatory factors such as TNF-α, IL-1β, and IL-6, as well as macrophage-induced IFN-β secretion, by suppressing STING expression. It also inhibits NF-κB phosphorylation, thereby alleviating excessive inflammatory responses [194]. Therefore, modulating the STING pathway through diverse microbial interventions may offer differentiated microbial intervention strategies and potential therapeutic candidates for precision-targeted treatment of distinct diseases, including tumors and excessive inflammation.

The inclusion of such STING modulators in this review is based on their clear research value and theoretical significance, not mature clinical evidence. STING inhibitors, including UM-200, ISD017, natural product derivatives such as Obtusilactone B and EGCG, and traditional Chinese medicine preparations which are mostly in preclinical stages, fill the gaps in STING-related disease targeted therapy and enrich low-toxic, multi-target modulator research. Additionally, novel strategies including STING ADCs, exosome and nanotechnology-based delivery, combination therapy and microbial intervention, which are limited to preclinical or early clinical stages, address traditional STING regulation pain points, offer new clinical transformation targets, and lay a theoretical foundation for effective STING-targeted drugs—this is the core reason for their inclusion. In summary, significant progress has been made in the structural and mechanistic research of STING, revealing its diverse roles in various diseases beyond its traditional involvement in innate immunity; however, the process of STING-targeted drug discovery remains unsatisfactory. Drawing on valuable insights from previous clinical trials of STING agonists, we eagerly anticipate the outcomes of upcoming clinical trials of STING inhibitors, as the continuous advancement of basic, clinical and translational research will undoubtedly promote the development of effective drugs to address unmet medical needs.

**Table 1:** Overview of STING agonists, their mechanisms, and liver diseases relevance

Drug names	Target cells	Effection/application on diseases	Mechanism	References
3′3′-cAIMP	Macrophage, DCs	Suppress Viral hepatitis and HCC	Secret IFN-Ⅰ and recruit T cells and NK cells to mount immune responses	[67]
cGAMP	Macrophage	Alleviate HCC	Upregulate proinflammatory cytokines, chemokines, and IFN-Ⅰ genes	[143]
	DCs	Ameliorate Melanoma		[195]
DMXAA	Macrophage	Ameliorate Viral hepatitis and CHB	Induce IFN-I, mediating antiviral activity	[40]
CMA	Macrophage	Suppress Viral hepatitis	Induce IFN-I, mediating antiviral activity	[145]
ADU-S100	DCs, Macrophage	AmeliorateCancer, and Atopic Dermatitis	Upregulation of proinflammatory cytokines and IFN-Ⅰ genes.	[153]
MK-1454	DCs	Ameliorate Cancer	Activate NF-κB and IRF3 pathways	[196]
TAK-676	DCs, NK cell, T cell	Ameliorate Cancer	Agonist-induced phosphorylation of TBK1 and STING, along with subsequent IRF3 activation	[197]
c-di-AMP	DCs	Ameliorate Viral hepatitis and Cancer	Induce transcription of IFN-Ⅰ and downstream immune response-related genes	[144]
Fe/Mn nanovaccine	DCs, Macrophage, T cell	Ameliorate HCC	Stimulate IFN-Ⅰ production and promote DCs maturation	[178]
MSA-2	Macrophage, APCs	Ameliorate HCC and CRC	Activate the TBK1-IRF3, promoting the secretion of IFN-β and pro-inflammatory factors	[180]
pH-sensitive nanoparticles	DCs, macrophage	Alleviate Melanoma and CRC	Activate the TBK1-IRF3 pathway, promoting IFN-Ⅰ secretion	[27]
Teniposide in combination with hyperbaric oxygen (HBO)	DCs	Ameliorate HCC	Enhance IFN-I and NF-κB signaling, activate DCs and T cells, and improve the response rate to PD-1 antibody	[86, 190]
*Lactobacillus rhamnosus GG (LGG)*	DCs	Ameliorate Inflammatory colitis	Promote IL-10 expression and IFN- Ⅰ expression	[192]
*Mycobacterium paragordonae (M. paragordonae)*	DCs, NK cell	Enhances Tumor Immunotherapy and Anti-Pathogen Immunity	Secrete c-di-GMP to activate STING via the IRE1α/XBP1s pathway	[193]
CHK1 inhibitor	DCs	Ameliorate CRC	Activate the cGAS-STING-TBK1 pathway via dsDNA accumulation^1^	[198]
Manganese-Protein Nanoparticle (Mn^2+^)	NK cell	Ameliorate Liver Fibrosis	Enhance immune clearance	[183]
Low-dose sorafenib with STAT3 knockdown	DCs	Ameliorate HCC	Enhance IFN-Ⅰ production and Activate CD8 T cells and NK cells	[78]

^1^Although in some contexts it only induces TBK1 phosphorylation without IRF3 activation while suppressing CGAMP-mediated STING signaling

**Table 2:** Overview of STING antagonists, their mechanisms, and liver disease relevance

Drug names	Target cells	Effection/application on diseases	Mechanism	References
C-176	Macrophage, Neutrophils	Alleviate Liver injury	Bind to STING and inhibit its activity	[162]
H-151	Macrophage, Neutrophils	Alleviate HIRI	Bind to STING and block its activation-induced palmitoylation	[163]
Palbociclib	APC, T cell	Ameliorate Autoimmunity and chronic inflammation	Binding to domain G166 and Tyr167 to disrupt CDN binding, STING dimerization, and TBK1 complex formation	[199]
ErTao decoction	Macrophage	Ameliorate Liver Fibrosis	Suppress M1 macrophage polarization, reduce inflammation, and inhibit the activation of HSC	[165]
Lingguizhugan decoction	Macrophage	Improve MASLD	Inhibit the activation of TBK1-NF-κB pathway and reduce IFN-β and TNF-α	[166]
Total tanshinones	Macrophage	Ameliorate Acute liver injury and Autoimmune disease	Inhibit STING and IRF3 phosphorylation	[169]
miR-24-3p	Macrophage	Ameliorate MASH	Inhibit TBK1-IRF3 signaling, promote M2 polarization, reduce the release of inflammatory factors	[176]
ISD017	Acts on STING-expressing cells	Ameliorate SLE	Interact with STIM1 to block the critical translocation of STING from the endoplasmic reticulum to the Golgi apparatus	[160]
PD-L1	T cell	Ameliorate Cancer	Reverse the immune escape caused by upregulation of tumor cell PD-L1 following STING activation	[186]
Bifidobacterium triple viable powder	Macrophage	Ameliorate MASLD and Chronic colitis	Suppress pro-inflammatory cytokine and IFN-β secretion	[194]

## Conclusion

As a central signaling axis linking innate and adaptive immunity, the cGAS-STING pathway plays an irreplaceable bidirectional regulatory role in maintaining hepatic immune homeostasis and in the progression of inflammatory liver diseases, metabolic liver diseases, and HCC. The conclusions presented in this paper regarding the interactions between immune cells, such as macrophages, dendritic cells, natural killer cells, and T cells, and the cGAS-STING pathway are based on a systematic review and synthesis of published research evidence from liver tissue and liver disease models, and are supported by clear and reliable direct experimental data. However, regarding NKT cells, neutrophils, B cells, eosinophils, mast cells, and basophils, there is currently a lack of direct research evidence from liver disease models; only relevant reports from non-liver models can be referenced. This paper cites such literature solely to outline the potential direct or indirect regulatory associations between the aforementioned cells and the cGAS-STING pathway, objectively identify existing research gaps in this field, and provide a reference basis for future exploration of relevant mechanisms.

Both cell-intrinsic and extrinsic STING activation in immune cells can shape their biological functions. However, the ultimate biological effects of STING activation depend on the intrinsic properties of the immune cells involved, the stage of disease progression, the microenvironment, and the duration of stimulation. Whether endogenous or exogenous, STING activation primarily influences the behavior of immune cells and reshapes the microenvironment through cytokines; at different stages of disease progression, it may induce diametrically opposed immune responses in the corresponding immune cells, such as its dual roles in fibrosis and tumor progression. Given the complexity and plasticity of the hepatic immune microenvironment, the development of STING-targeted therapeutics faces substantial challenges. These include: deciphering the microenvironment-dependent regulatory networks underlying dynamic signaling cascades; achieving spatiotemporally precise targeting to balance immune activation and excessive inflammation; and overcoming clinical translation bottlenecks caused by insufficient target specificity and systemic toxicity. Therefore, it is critical to carefully evaluate the potential benefits and risks of STING agonism as a therapeutic strategy for certain liver diseases. Despite conflicting observations and context-dependent dual effects, accumulating evidence supports the STING pathway as a promising therapeutic target across diverse liver diseases.

Future studies should focus on dissecting the cell-specific regulatory patterns of the cGAS-STING pathway at different disease stages, developing intervention tools with improved spatiotemporal controllability and cell-type selectivity, and performing rigorous preclinical validation. This will provide critical theoretical and experimental support for clarifying the molecular mechanisms underlying hepatic immune-metabolic disorders and establishing precision immunotherapeutic strategies, ultimately accelerating the clinical translation of cGAS-STING pathway-targeted treatments.

## Data Availability

No datasets were generated or analysed during the current study.

## Abbreviations

2’3’-cGAMP: 2’-3’-cyclic GMP-AMP
CDNs: Cyclic dinucleotides
TREX1: The nucleic acid repair exonuclease 1
TRIM29: Tripartite motif containing 29
NLR: NOD-like receptor
ISG: Interferon-stimulated genes
PAMPs: Pathogen-associated molecular patterns
DAMPs: Damage-associated molecular patterns
TAMs: Tumor-associated macrophages
CX32: Connexin 32
DN stage: Double-negative stage
DP stage: Double-positive stage
SP stage: Single-positive stage
APCs: Antigen-presenting cells
STAT1: Signal transducer and activator of transcription 1
PD-L1: Programmed death-ligand 1
IDO1: Indoleamine 2,3-dioxygenase 1
IL-17A: Interleukin-17 A
3’3’-cAIMP: 3’3’-cyclic adenosine-inosine monophosphate
cGAMP: Cyclic GMP–AMP
DMXAA: 5,6-dimethylxanthenone-4-acetic acid
c-di-AMP: Cyclic diadenosine monophosphate
CHK1 inhibitor: Checkpoint kinase 1 inhibitor
HCC: Hepatocellular carcinoma
CHB: Chronic hepatitis B
CRC: Colorectal cancer
HSC: Hepatic stellate cell
STIM1: Stromal interaction molecule 1
SLE: Systemic lupus erythematosus
HIRI: Hepatic ischemia-reperfusion injury
